# Changes in immunological profile of allogeneic mesenchymal stem cells after differentiation: should we be concerned?

**DOI:** 10.1186/scrt488

**Published:** 2014-08-19

**Authors:** Paul Lohan, Cynthia M Coleman, J Mary Murphy, Matthew D Griffin, Thomas Ritter, Aideen E Ryan

**Affiliations:** Regenerative Medicine Institute, College of Medicine, Nursing and Health Sciences, National University of Ireland, Galway, Ireland; Discipline of Pharmacology and Therapeutics, College of Medicine, Nursing and Health Sciences, National University of Ireland, Galway, Ireland

## Abstract

Mesenchymal stem cells (MSCs) are an adult stromal cell population possessing potent differentiation capacity and a potential for use across major histocompatibility complex barriers. Although allogeneic MSCs have potent immunosuppressive properties, evidence also suggests that they elicit a weak allogeneic immune response. However, the effect of induced differentiation on the immunosuppressive ability and immunogenicity of allogeneic MSCs is a potential obstacle when applying MSCs in tissue replacement therapies. These concerns will be explored in this review, with particular emphasis on changes in the cell surface expression of immunogenic markers, changes in the secretion of immunosuppressive molecules and *in vivo* functional benefits of the cell therapy. We review the literature from a translational point of view, focusing on pre-clinical studies that have utilised and analysed the effects of allogeneic immune responses on the ability of allogeneic MSCs to regenerate damaged tissue in models of bone, heart and cartilage defects.

## Introduction

Organ transplantation, as a medical procedure to replace a damaged or defective organ, has been performed for over 100 years. Numerous organs and tissues are now routinely transplanted, including heart, kidney, islets, liver, lung, cornea and skin [1]. The immune response to, and consequent rejection of, allogeneic organ and tissue grafts has always been a major issue and numerous strategies have been developed to inhibit immune responses, including irradiating the recipient, immunosuppressive drugs and, more recently, cellular therapies [1–3]. Despite the effectiveness of these treatment modalities, many transplanted organs still undergo acute and chronic immune-mediated rejection episodes that have drastic consequences for the survival and general health of the patient [4].

Because of the immunological difficulties associated with allogeneic transplantation, mesenchymal stem cells (MSCs) have the potential to be an attractive tissue replacement therapy for a number of reasons. MSCs are multipotent cells that can be readily isolated from a number of adult tissues, including bone marrow, umbilical cord blood, adipose tissue and placenta. They have been well documented to differentiate into osteogenic, adipogenic and chondrogenic lineages *in vivo*, with evidence also supporting an ability to differentiate into cardiomyocyte, endothelial, hepatocyte and neural lineages [2, 5–13]. Differentiated MSCs (dMSCs) could be used in tissue engineering- and regenerative medicine-based approaches to treat a number of conditions.

In addition to their differentiation capacity MSCs are defined by their ability to suppress immune responses in addition to eliciting a weak cellular and humoral allogeneic immune response (here referred to as low immunogenicity) [2, 14]. These immune properties of MSCs can be attributed to a combination of low expression of immunogenic cell surface molecules and secretion of several immunosuppressive molecules. MSCs from numerous sources and species have been shown to express low levels of major histocompatibility complex class I (MHCI) proteins on their cell surface as well as low levels of co-stimulatory molecules, such as CD80 and CD86; additionally, in the resting state, MSC express no major histocompatibility complex class II (MHCII) proteins [5–7, 9, 14, 15]. These properties contribute to the so-called hypo-immunogenicity of MSCs. The immunosuppressive ability of MSCs is attributed to their secretion of several immunomodulatory molecules, such as prostaglandin E2 (PGE2), nitric oxide, indoleamine-2,3 dioxygenase and tumour necrosis factor-α stimulated gene-protein 6, that can interfere with proliferation, activation and effector function of many cells of the immune system, including CD4+ and CD8+ T cells, natural killer cells, B cells and antigen presenting cells [5, 6, 16–21]. MSCs are also capable of increasing the population of regulatory T cells [22]. This immunosuppressive ability has been reported to be cell contact dependent and induced *in vivo* by activation of the cells through encountering inflammatory cytokines such as IFN-γ [21, 23].

Allogeneic MSCs from young healthy donors are an attractive source of regenerative cells for the treatment of degenerative diseases with an inflammatory component. As MSCs possess potent immunomodulatory properties and an ability to differentiate into several lineages, there is potential for allogeneic 'off the shelf' tissue engineering solutions using these cells. These treatment options would significantly decrease costs, reduce the number of procedures patients must undergo and provide cells from young healthy donors that may show higher efficacy than cells from aged individuals [24]. Although evidence exists to suggest MSC immunomodulatory properties may differ depending on the tissue from which they are sourced [25] or by contact with serum [26], there is no information directly comparing the immune profile of allogeneic dMSCs from different sources or after contact with serum. These important issues should be investigated in future studies. With the increasing number of clinical trials utilising allogeneic MSCs for acute and chronic diseases, a comprehensive understanding of the impact of differentiation on the immunological profile of MSCs is essential.

Clinical application of allogeneic MSCs could take the form of *ex vivo* differentiation of the MSCs followed by administration to the damaged area or administration of undifferentiated MSCs that subsequently undergo differentiation *in situ.* Highly prevalent acute and chronic diseases for which current treatments are suboptimal, such as myocardial infarction (MI; prevalence of 3.2% of US population in 2009) and osteoarthritis (OA; 27 million people in US with clinical grade OA), are potential targets for allogeneic dMSC therapy. In the context of available evidence [12, 13] the focus of this review will be on immune responses to and therapeutic potential of allogeneic MSCs differentiated into osteogenic, chondrogenic and cardiomyocyte lineages [27, 28].

## Allogeneic mesenchymal stem cells in bone regeneration

Allogeneic MSCs have been proposed for use in the repair of critical size bone defects as well as a treatment for osteogenesis imperfecta (OI) [11, 29–32]. With the field moving increasingly towards allogeneic cell therapeutic modalities [33], for reasons alluded to earlier, the immunogenic potential of donor-derived *ex vivo* osteogenically primed or *in vivo* osteogenically differentiated MSCs must be highlighted.

A number of pre-clinical studies focusing on the functional benefits of allogeneic MSC implantation in bone regeneration have produced contrasting results on reparative outcomes, as can be seen in Table 1. Kang and colleagues [31] demonstrated that implantation of undifferentiated allogeneic or autologous MSCs comparably supported the development of *de novo* bone without lymphocytic infiltration. Similarly, Liu and colleagues [7] and Arinzeh and colleagues [32] implanted *ex vivo* osteogenically differentiated cells *in vivo* in a leporine and canine model, respectively, and found that these cells incorporated into the host tissue, functioned as osteoblasts and resided *in situ* for at least 28 days without overt signs of rejection such as hypercellularity. In contrast to these data, however, it has been reported by others that implanted allogeneic MSCs require immunosuppressive treatment to survive and differentiate *in vivo,* and are rapidly cleared by infiltrating immune cells in the absence of such immunosuppression [34, 35]. Additionally, expression of immunogenic molecules such as Swine leukocyte antigen-I (SLA-I) on differentiating allogeneic porcine MSCs was shown to reduce the *in vivo* efficacy of the treatment when compared to allogeneic MSCs that had SLA-I knocked down [36]. Wang and colleagues [37] showed that *in vitro* cultured ovine MSCs maintain MHCI expression at similar levels before and after osteogenic differentiation, while osteogenic differentiated ovine and leporine MSCs are negative for MHCII and fail to stimulate proliferation in allogeneic leukocyte proliferation assays [7, 37]. However, these MHCII-negative cells retain a rich deposit of MHCII mRNA in the cytoplasm that is rapidly translated and presented on the cell surface upon stimulation with IFN-γ [38]. This has been confirmed *in vivo* where implanted allogeneic osteogenically primed MSCs upregulate surface expression of MHCII*,* pointing to a potentially increased immunogenic phenotype when these cells are exposed to an inflammatory environment [7].

**Table 1:** **Summary table of differentiated allogeneic MSC in bone regeneration models**

Paper	Model	*In vitro*immunogenicity	*In vitro*immunosuppressive ability	*In vivo*engraftment	*In vivo*immune marker expression	*In vivo*functional benefits	*In vivo*cellular response	*In vivo*antibody response
Wang *et al.* [37]	*In vitro* assessment of osteogenically differentiated MSCs from swine	No increase in SLAI. Slight increase in SLAII. Osteogenically differentiated MSCs were equivalently immunogenic as undifferentiated MSCs	Osteogenically differentiated and undifferentiated MSCs displayed equivalent immunosuppressive ability	NT	NT	NT	NT	NT
Liu *et al.* [7]	Osteogenically differentiated rabbit MSCs; ectopic transplant	Osteogenically differentiated MSCs lacked surface MHCII. No difference in *in vitro* immunogenicity or susceptibility to cytotoxic lysis	No difference in immunosuppressive ability between differentiated and undifferentiated MSCs	NT	Upregulation of MHCII on implanted dMSCs	Implanted allogeneic dMSCs produced osteonectin and osteopontin *in vivo*	No increased rejection of allogeneic skin grafts after dMSC treatment	NT
Le Blanc *et al.* [38]	*In vitro* assessment of human dMSCs	No significant increase in *in vitro* immunogenicity despite increase in HLAI cell surface expression	Osteogenically differentiated MSCs retained their immunosuppressive ability *in vitro*	NT	NT	NT	NT	NT
Kang *et al.* [31]	Allogeneic MSCs in allogeneic bone matrix to radial defect in New Zealand White rabbit	NT	NT	Both autologous and allogeneic MSCs were capable of facilitating bone regeneration	NT	Initial bone quality index equivalent between autologous and allogeneic MSCs, but significantly higher in autologous MSC-treated group after 12 weeks	No cellular infiltrate observed	NT
Arinzeh *et al.* [32]	Scaffold loaded allogeneic MSCs to canine critical sized femoral defect	**NT**	NT	Implanted allogenic MSC detected up to 16 weeks	NT	Bone regeneration observed at 16 weeks	No lymphocytic infiltration observed	No alloantibodies detected
Kotobuki *et al.* [34]	Lewis MSCs on hydroxyapatite scaffolds to F344 rats	NT	NT	NT	NT	Immunosuppression was required for *in vivo* osteogenic differentiation of allogeneic MSCs	Possible infiltration of inflammatory cells	NT
Chatterjea *et al.* [35]	Allogeneic MSC-derived osteoprogenitors in ectopic rat model	NT	NT	NT	NT	Allogeneic osteoprogenitors require immunosuppression to form bone	T and B cell infiltration to allogeneic graft. Effects were mediated by immunosuppression	NT
Ren *et al.* [36]	MHCI knock-down MSCs in various animal models	NT	NT	NT	NT	MHCI knock-down MSC-treated animals showed better bone regeneration	Higher frequency of circulating activated lymphocytes in animals treated with wild-type MSCs	NT
Horwitz *et al.* [30]	OI patients who had previously received bone marrow transplants administered MSCs derived from the same donor	NT	NT	5 of 6 patients demonstrated MSC engraftment	NT	5 of 6 patients demonstrated markedly increased growth velocity	Cellular response to viral antigens in some patients	1 patient produced anti-FBS antibodies
Le Blanc *et al.* [29]	Allogeneic foetal liver-derived MSCs to foetus diagnosed with OI	NT	NT	Allogeneic dMSCs detected in bone biopsy at 9 months (up to 7.4%)	NT	Patient growth could be attributed to allogeneic MSC therapy	No memory response against donor undifferentiated MSCs	

Data related to immunological profile of MSCs both *in vitro* and *in vivo* are collated. dMSC, differentiated mesenchymal stem cell; FBS, foetal bovine serum; HLAI, human leukocyte antigen class I; MHCI, major histocompatibility complex class I; MHCII, major histocompatibility class II; MSC, mesenchymal stem cell; NT, not tested; OI, osteogenesis imperfecta; SLAI, swine leukocyte antigen class I; SLAII, swine leukocyte antigen class II.

In summary, contrasting pre-clinical outcomes in relation to the immunogenicity of osteogenically primed MSCs have been reported, indicating the need for more powerful pre-clinical studies to be completed before a definitive conclusion on the potential of allogeneic MSCs for bone tissue regeneration can be made.

Most importantly, the osteo-integration, differentiation and immune acceptance of transplanted allogeneic MSCs in humans must be considered. Allogeneic MSCs have begun to be administered clinically to treat OI, necessitating the homing, integration and function of allogeneic cells within the host. In contrast to the pre-clinical studies outlined above, MSCs were administered intravenously. Horwitz and colleagues [30] treated six patients with two transfusions, initially 1 million cells/kg and then 5 million cells/kg 8 to 21 days later. No patient exhibited symptoms of immune rejection, while five of the six children demonstrated persistent engraftment of the transplanted cells within the host tissue. Engraftment, even at low levels, resulted in clinically significant improvement in mineralized tissue deposition. However, it is not clear from this study if the engrafted cells differentiated into osteogenic lineages [30]. Le Blanc and colleagues [29] later transplanted allogeneic human foetal liver MSCs at 10 weeks gestation *in utero* to a foetal recipient diagnosed with OI. Post-natal observation at 9 months confirmed the engraftment and functional differentiation of these transplanted cells via expression of osteocalcin and bone sialoprotein. There was no immunological reaction, either *in vivo* or in *ex vivo* re-stimulation assays for up to 10 years. Over time, however, allogeneic cells could no longer be detected and the functional benefits observed in the months after treatment were lost. A second infusion of the same donor cells was administered, which was shown not to elicit an immune response, and the patients’ growth was restored [39]. Although direct comparison of results of pre-clinical and clinical studies is complicated by the use of different administration routes, the data gathered thus far indicate that human allogeneic transplanted MSCs are capable of homing to osseous tissue, integrating with the host and maturing into functional osteoblasts without systemic immune rejection.

## Allogeneic mesenchymal stem cells in cardiac regeneration

MI occurs as a result of loss of blood flow to an area of the myocardium, resulting in ischemia and degeneration of the muscle, leading to loss of cardiac function and potentially death [40]. MSCs have therapeutic potential in MI due to their secretion of paracrine regenerative and pro-angiogenic factors, their homing ability and their capacity to differentiate into cardiomyocytes, smooth muscle and vascular endothelial lineages [40, 41]. However, due to the fact that MI is a sudden event and an immediate treatment may be necessary for an effective outcome, allogeneic MSC therapy may be the ideal treatment for MI [6].

As allogeneic *in vitro* cultured MSCs administered to the area of an MI have the capacity to differentiate to endothelium, smooth muscle and cardiac muscle, a thorough understanding of the host immune response to these newly differentiated cells is required before their translation to the clinic. To date, allogeneic undifferentiated MSCs have been administered in several pre-clinical models of MI. While many of these studies did not examine immunological parameters, as can be seen in Table 2, they showed that MSCs are capable of engraftment into the damaged myocardium, specifically in the infarct and border zones [42–46]. These engrafted cells have been shown, in several separate studies, to express markers of cardiac muscle, smooth muscle and vascular endothelium, such as MF-20, troponin I, vascular endothelium growth factor and von Willebrand factor [6, 40, 44–46]. In addition to expressing markers of myocardium, 5-azacytidine-treated MSCs have also been shown to upregulate expression of immunogenic MHCI and MHCII molecules. When these pre-treated allogeneic MSCs were administered to an infarcted myocardium, they were recognised by the immune system and quickly cleared from the area with an accompanying loss of beneficial effects [47].

**Table 2:** **Summary table of differentiated allogeneic MSC in myocardial regeneration models**

Paper	Model	*In vitro*immunogenicity	*In vitro*immunosuppressive ability	*In vivo*engraftment	*In vivo*immune marker expression	*In vivo*functional benefits	*In vivo*cellular response	*In vivo*antibody response
Xia and Cao [47]	Balb/C cardiomyocyte dMSCs to C57/BL6 mouse MI model	Increased MHCI and MHCII expression on cardiomyocyte dMSCs	NT	Both undifferentiated and differentiated allogeneic MSCs engrafted. Over 4 weeks dMSCs were cleared quicker than undifferentiated	NT	Both differentiated and undifferentiated MSCs improved function at 2 weeks over controls; however, by 4 weeks benefit due to dMSCs was lost	CD4+ and CD8+ infiltration in both undifferentiated and differentiated groups; significantly more in differentiated	NT
Huang *et al.* [6]	Wistar rat (allogeneic) or Lewis (syngeneic) MSCs to Lewis rat MI model	MHCIa upregulated and MHCIb downregulated after *in vitro* differentiation. MHCII and CD86 co-expressed by dMSCs. Increased susceptibility to cytotoxic lysis	NT	Significantly more undifferentiated MSCs than dMSCs were engrafted at day 7	Engrafted dMSCs co-expressed MHCI or MHCII with differentiation markers	Allogeneic MSC-treated animals displayed loss of functional benefit over time compared to syngeneic MSC-treated animals	Leukocyte infiltration into allogeneic MSC-treated hearts	Allo-antibodies produced against differentiated but not undifferentiated MSCs
Dhingra *et al.* [18]	Wistar MSCs to Lewis rat MI	dMSCs more susceptible to cytotoxic lysis	MSCs lose ability to secrete PGE2 as they differentiate, which results in reduced ability to induce Tregs	MSCs were eliminated by 5 weeks; some remained engrafted after PGE2 augmentation	NT	Improvement noted, but this was significantly less than if PGE2 was co-administered with allogeneic MSCs	Increased CD8+ T-cell infiltration in dMSC-treated hearts, which could be rescued by PGE2	Allo-antibodies produced against dMSCs, which could be reduced by PGE2
Amado *et al.* [43]	Allogeneic porcine MSCs to porcine MI	NT	NT	Reported 42.4 ± 15% engraftment at 8 weeks. Labelled engrafted cells co-expressed differentiation markers	NT	Significant improvement after 8 weeks	NT	NT
Makkar *et al.* [44]	Allogeneic porcine MSCs to porcine heart 1 month after MI	NT	NT	Engrafted cells detected 2 months after injection	NT	No further deterioration in treated group compared to control	NT	NT
Perin *et al.* [45]	Allogeneic canine MSCs to canine MI model delivered either intra-coronarily or transendocardially	NT	NT	Engrafted cells detected 14 days after administration	NT	Transendocardially delivered allo-MSCs provided a functional benefit	NT	NT
Quevedo *et al.* [42]	Allogeneic porcine MSCs to porcine MI	NT	NT	Engrafted cells detected at 84 days co-expressing differentiation markers	NT	Improved cardiac function compared to control group	NT	NT
Dai *et al.* [46]	Allogeneic ACI rat MSCs to Fischer rat MI	NT	NT	7 of 7 hearts at 6 months showed engrafted MSCs that co-expressed myocardium markers	NT	Improved LVEF at 4 weeks in allogeneic MSC-treated rats compared to control; effects were lost by 6 months	NT	NT

Data related to immunological profile of MSCs both *in vitro* and *in vivo* are collated. dMSC, differentiated mesenchymal stem cell; LVEF, left ventricular ejection fraction; MHCI, major histocompatibility complex class I; MHCII, major histocompatibility class II; MI, myocardial infarction; MSC, mesenchymal stem cell; NT, not tested; PGE2, prostaglandin E2; Tregs, regulatory T cells.

Several reports have indicated that allogeneic undifferentiated MSC administration to infarcted myocardium results in improved recovery of cardiac functional parameters such as left ventricular ejection fraction at early time points up to 3 months after administration [6, 44–48]. However, as pre-differentiated MSCs have been shown to become strongly immunogenic, a major concern is that these administered allogeneic undifferentiated MSCs may differentiate *in situ* and develop a more immunogenic phenotype that could result in immune cell infiltration in the heart, clearance of the cells and loss of the functional benefits of the therapy. In order to determine the immunological effect of *in situ* differentiation on allogeneic MSCs, it is necessary to follow the consequences of allogeneic MSC therapy, both immunologically and functionally, for up to a year [49].

In a rat MI model undifferentiated allogeneic MSCs were shown to engraft, provide functional improvement for up to 3 months and differentiate into vascular endothelium, smooth muscle and cardiomyocytes. Huang and colleagues [6] showed that these cells elicit an allogeneic antigen-specific immune response, demonstrated by the presence of specific anti-donor antibodies directed against differentiated but not undifferentiated MSCs at 5 weeks post-administration that was accompanied by elimination of allogeneic cells at the same time-point. When the functional benefits of the therapy were determined, there was no difference between syngeneic and allogeneic MSC treatment at 3 months post-administration; however, after 6 months allogeneic MSC-treated animals had significantly reduced cardiac function compared to syngeneic MSC-treated animals [6]. Follow-up studies indicate that the rejection of allogeneic MSCs might be facilitated by the loss of PGE2 expression in the cells as they differentiate [5, 18, 49]. PGE2 is essential for expression of chemokines such as CCL5 and CCL12 that attract T cells to the MSCs and result in an increased proportion of regulatory T cells [18]. It was found that when allogeneic MSCs were administered to an MI together with extracellular PGE2 the survival of the cells was maintained, rejection was prevented and the functional benefits of the therapy were restored [18].

Allogeneic MSCs have been tested in early phase clinical trials of MI, although it was not determined if these cells differentiated *in situ.* No significant adverse events were observed up to 12 months after administration and cell-treated patients showed a significant increase in left ventricular ejection fraction compared to placebo, which was maintained throughout the 12-month observation period. Additionally, a trend towards improved cardiac remodelling was observed in cell-treated patients [50]. However, longer follow-up times will be required to determine if the benefit of allogeneic MSC therapy for MI can be maintained in the long-term and also to determine any additional immunological effects if a second cell administration is required.

## Allogeneic mesenchymal stem cells in cartilage regeneration

OA is a degenerative disease characterized by articular cartilage loss and synovial inflammation. While some regeneration of cartilage is possible in childhood, the regenerative capacity is lost with time and is nearly completely absent after 60 years of age [51]. The use of differentiated allogeneic MSCs is envisioned based on their ability to differentiate into cartilage [52], their ability to modulate inflammation by the release of anti-inflammatory factors [16, 19–21] and their low levels of MHC and co-stimulatory proteins [14]. Prior to their clinical use, and similar to the use of allogeneic MSCs for bone defects and cardiac repair, a comprehensive understanding of the allogeneic immune response to chondrogenically differentiated allogeneic MSCs is crucial for elucidating the success of stem cell-based cartilage repair *in vivo*. A small number of studies (outlined in Table 3) have addressed the impact of chondrogenic differentiation on the immunosuppressive ability of allogeneic MSCs from different sources. Although Zheng and colleagues [53] showed that dMSCs retain their ability to suppress allogeneic immune responses in a model of rheumatoid arthritis, other studies have shown an altered ability to suppress immune responses *in vitro* [5, 54]; Chen and colleagues [54] showed that chondrogenically differentiated MSCs, in contrast to undifferentiated allogeneic MSCs, were unable to suppress dendritic cell function. On the contrary, they showed that dMSCs induced dendritic cell maturation and the human peripheral blood leukocyte-stimulating and cytotoxicity-inducing effects of MSCs increased between four- and eight-fold in differentiated cultures compared to undifferentiated MSCs. Similarly, we showed significant loss of ability to suppress activated CD4+ and CD8+ T cells, which was accompanied by inhibition of PGE2 and nitric oxide [5].

**Table 3:** **Summary table of differentiated allogeneic MSC in cartilage regeneration**

Paper	Model	*In vitro*immunogenicity	*In vitro*immunosuppressive ability	*In vivo*engraftment	*In vivo*immune marker expression	*In vivo*functional benefits	*In vivo*cellular response	*In vivo*antibody response
Ryan *et al.* [5]	Allogeneic rat chondrogenically differentiated MSCs implanted subcutaneously	Increased T-cell proliferation and activation. Increased susceptibility to allo-specific cytotoxic lysis. Granzyme B + CD8+ T cells generated against dMSCs	Immunosuppressive ability lost after differentiation. PGE2 and NO secretion significantly reduced	Extensive cellular infiltration leading to graft damage	NT	NT	CD3+ and CD68+ immune cell infiltration. Local and systemic cellular memory response to dMSCs; this response only seen locally and without encapsulation in undifferentiated MSCs	Increased anti-donor antibody levels. Th1 type antibody response
Zheng *et al.* [53]	Human RA patients' T cells with allogeneic chondrogenically differentiated MSCs	No collagen II-specific T-cell proliferation to dMSCs	dMSCs could suppress allogeneic T-cell proliferation and activation.	NT	NT	NT	NT	NT
			dMSCs could suppress CD4+ and CD8+ inflammatory cytokine production.					
			dMSCs and undifferentiated MSCs secreted similar TGFβ1 levels					
Technau *et al.* [55]	*In vitro* assessment of human chondrogenically differentiated MSCs	dMSCs stained positive for HLA-ABC and HLA-DR.	NT	NT	NT	NT	NT	NT
		dMSCs secreted IFNγ						
Chen *et al.* [54]	*In vitro* assessment of rat chondrogenically differentiated MSCs	Upregulation of CD80 and CD86	NT	NT	NT	NT	NT	NT

Data related to immunological profile of MSCs both *in vitro* and *in vivo* are collated. dMSC, differentiated mesenchymal stem cell; HLA-ABC, human leukocyte antigen ABC; HLA-DR, human leukocyte antigen DR; IFN-γ, interferon-γ; MSC, mesenchymal stem cell; NO, nitric oxide; NT, not tested; PGE2, prostaglandin E2; RA, rheumatoid arthritis; TGFβ1, transforming growth factor β1.

The limited number of studies [5, 54, 55] addressing the important issue of immunogenicity hinder our understanding of the consequences of allogeneic MSC differentiation on therapeutic efficacy in functional models of cartilage repair. Chondrogenic differentiation has been shown to increase the immunogenicity of MSCs and many studies have shown increases in the expression of MHCI, MHCII, CD80 and CD86 [5, 54, 55]. We recently addressed *in vivo* immunogenicity and observed enhanced T-cell and innate immune responses following subcutaneous implantation of chondrogenically differentiated fully allogeneic rat MSC [5]. Using an *ex vivo* re-stimulation assay, detectable sensitized T-cell responses were seen in animals 6 weeks post-implantation with chondrogenically differentiated MSCs, which was accompanied by increased generation of donor-specific antibodies. Interestingly, while these responses were prevented by encapsulation in the case of undifferentiated allogeneic MSCs, encapsulation did not prevent immune responses generated against chondrogenically differentiated MSCs [5]. These findings are also relevant for spontaneous differentiation of allogeneic undifferentiated MSCs *in vivo* and may explain discrepancies between studies using undifferentiated MSCs as a therapeutic in models of OA. In fact, a recent study showed that undifferentiated fully allogeneic MSCs lose their immunosuppressive properties and this compromises their ability to influence the course of collagen-induced arthritis [56].

## Conclusion

Due to the inherent lack of native regeneration in bone, heart and cartilage tissues, regenerative medicine-based approaches hold great therapeutic promise [6, 30, 51]. MSCs, as regenerative cells, are attractive for therapeutic use in these diseases due to their ease of isolation and culture and their *ex vivo* and *in situ* differentiation capacities. However, cell therapy using autologous cells is a time-consuming, expensive process with other disadvantages such as donor site morbidity and quality issues with using cells from aged patients [6, 31]. For these reasons allogeneic MSC therapy in the context of regenerative medicine must be investigated pre-clinically with an ultimate objective of translating such therapies to the clinic.

**Figure 1:**
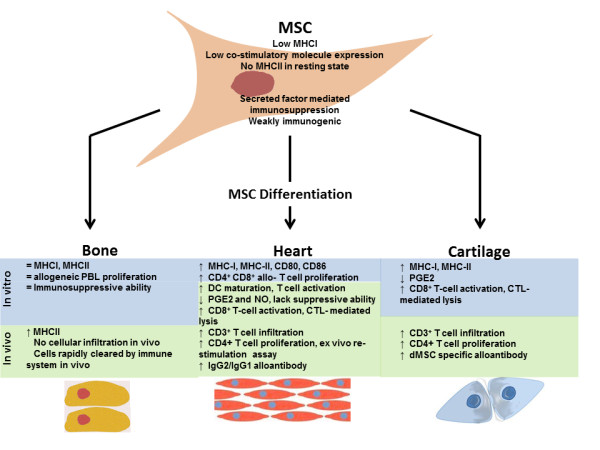
**The impact of osteogenic, chondrogenic and myocardial differentiation on immunogenicity of allogeneic mesenchymal stem cells.** Changes and responses to allogeneic mesenchymal stem cells (MSCs) as they differentiate *in vitro* (blue shaded areas) or *in vivo* (green shaded areas) are represented. General immunological characteristics of MSCs are represented along with documented changes that take place as they differentiate into osteogenic, chondrogenic and cardiomyocyte lineages. Changes to relevant molecules are indicated with up arrows representing an increase, down arrows representing a decrease and an equals sign representing no change in the indicated parameter. CTL, cytotoxic T lymphocyte; DC, dendritic cell; dMSC, differentiated MSC; Ig, immunoglobulin; MHCI, major histocompatibility complex class I; MHCII, major histocompatibility complex class II; MSC, mesenchymal stem cell; NO, nitric oxide; PBL, peripheral blood leukocyte; PGE2, prostaglandin E2.

Important issues raised during this review focus upon the potential changes to the potent immunomodulatory properties of MSCs that occur after differentiation of these cells. While some of the literature appears contradictory, there is evidence that the secretory profile of MSCs is altered as they differentiate [5, 18]. Although this requires a more thorough analysis of the full secretome of MSCs differentiated into various lineages, there is already convincing experimental evidence that differentiation-associated reduction in the secretion of immunomodulatory factors such as PGE2 may have adverse effects on the survival of the allogeneic graft *in vivo* and ultimately the reparative capacity of allogeneic MSCs. Recognition of allogeneic cells and the subsequent immune response is a critical problem for solid organ transplantation mediated by the presence of immunologically relevant surface proteins such as MHCI, MHCII and co-stimulatory molecules on the transplanted cells [57]. Many of the studies we have reviewed here point to an increase in expression of these immunogenic molecules on the cell surface of MSCs as they differentiate (Figure 1) [5–7]. Nevertheless, other studies have provided evidence suggesting no increased immunogenicity or MHC upregulation for other lineages, such as hepatocytes and neurons [12, 13]. To date, however, insufficient pre-clinical data are available, suggesting further studies are required to conclusively show changes in immune responses after differentiation into these lineages.

As evidenced in the majority of studies reviewed here, heightened immune responses may be induced by dMSCs *in vivo.* Several potential strategies could conceivably be employed to reduce the immunogenicity of the cells or increase their immunosuppressive ability. Such approaches could include addition of PGE2 [18] to restore the immunosuppressive ability, which has shown some promise in pre-clinical studies. In recent years pre-treatment of MSCs with inflammatory cytokines such as IFN-γ has shown that these pre-treated MSCs have ncreased immunosuppressive ability *in vivo* [58]. Pre-treating undifferentiated MSCs or dMSCs prior to implantation may improve the immunosuppressive function of these cells *in vivo.* Encapsulation of dMSCs in semi-permeable membranes to shield the cells has the potential to protect cells from immune cell and complement attack, as has been previously described in the case of pancreatic islets [59]. Some data also show promise for such techniques where dMSCs were implanted *in vivo* [5]. Other potential approaches to improve allogeneic dMSC transplant survival *in vivo* could be achieved by genetic knockdown of immunogenic molecules such as MHCI [36, 60].

Further comprehensive analyses of the functional relevance of increased expression of MHCI, MHCII and co-stimulatory molecules on dMSCs *in vivo* need to be undertaken, since not all studies to date are in agreement on the issue of immunogenicity. In addition, a thorough assessment of the impact of allo-immune responses on therapeutic efficacy of dMSCs in fully allogeneic models needs to be undertaken. This is especially true in the case of chondrogenically differentiated allogeneic MSCs where further pre-clinical experiments to assess the utility of these cells in fully allogeneic cartilage regeneration models are required.

What is clear is that several issues need to be addressed before translation of allogeneic dMSC therapies to the clinic: first, whether the allogeneic immune response hinders the therapeutic efficacy of these cells and how we interpret these findings; second, the long-term survival and functional state of dMSCs *in vivo*; third, the paracrine effects of dMSCs *in vivo*; fourth, whether modification of allogeneic dMSCs prior to transplantation is required; and fifth, whether concomitant administration of immunosuppressive drugs is required [11]. The answers to these questions will undoubtedly unfold in due course.

## Abbreviations

dMSC: Differentiated mesenchymal stem cell
IFN: Interferon
MHCI: Major histocompatibility complex class I
MHCII: Major histocompatibility complex class II
MI: Myocardial infarction
MSC: Mesenchymal stem cell
OA: Osteoarthritis
OI: Osteogenesis imperfecta
PGE2: Prostaglandin E2
SLA-I: Swine leukocyte antigen-I.
